# Extracellular Calcium-Dependent Modulation of Endothelium Relaxation in Rat Mesenteric Small Artery: The Role of Potassium Signaling

**DOI:** 10.1155/2015/758346

**Published:** 2015-10-04

**Authors:** Lise Hangaard, Peter B. Jessen, Dmitrii Kamaev, Christian Aalkjaer, Vladimir V. Matchkov

**Affiliations:** ^1^MEMBRANES, Department of Biomedicine, Health, Aarhus University, 8000 Aarhus, Denmark; ^2^Department of Biomedicine, University of Copenhagen, Copenhagen, Denmark

## Abstract

The nature of NO- and COX-independent endothelial hyperpolarization (EDH) is not fully understood but activation of small- and intermittent-conductance Ca^2+^-activated K^+^ channels (SK_Ca_ and IK_Ca_) is important. Previous studies have suggested that the significance of IK_Ca_ depends on [Ca^2+^]_out_. Also it has been suggested that K^+^ is important through localized [K^+^]_out_ signaling causing activation of the Na^+^,K^+^-ATPase and inward-rectifying K^+^ channels (K_ir_). Here we tested the hypothesis that the modulating effect of [Ca^2+^]_out_ on the EDH-like response depends on [K^+^]_out_. We addressed this possibility using isometric myography of rat mesenteric small arteries. When [K^+^]_out_ was 4.2 mM, relaxation to acetylcholine (ACh) was stronger at 2.5 mM [Ca^2+^]_out_ than at 1 mM [Ca^2+^]_out_. Inhibition of IK_Ca_ with TRAM34 suppressed the relaxations but did not change the relation between the relaxations at the low and high [Ca^2+^]_out_. This [Ca^2+^]_out_-dependence disappeared at 5.9 mM [K^+^]_out_ and in the presence of ouabain or BaCl_2_. Our results suggest that IK_Ca_ are involved in the localized [K^+^]_out_ signaling which acts through the Na^+^,K^+^-ATPase and K_ir_ channels and that the significance of this endothelium-dependent pathway is modulated by [Ca^2+^]_out_.

## 1. Introduction

The importance of the arterial endothelium for controlling vascular resistance is well-established [1]. Several factors are released from the endothelium and relax the adjacent smooth muscle cells. In addition to NO and prostaglandins, the endothelium is able to produce vasodilatation through a third pathway, which is particularly important in small arteries and arterioles [2]. The third pathway is the endothelium-dependent hyperpolarization (EDH) of smooth muscles, which is present after the inhibition of NO and prostaglandin production [3, 4]. The mechanisms proposed for EDH are direct spreading of hyperpolarizing current via myoendothelial gap junctions or release of diffusible factors(s) (EDHF(s)). The relative contribution of these two pathways and the nature of the EDHFs vary among species and vascular beds, blood vessel caliber, ageing, and diseases, as well as between different experimental conditions and laboratories [3, 5]. It is unlikely that a single factor accounts for EDHF and multiple diffusible factors, including K^+^, NO, HNO, epoxyeicosatrienoic acids, H_2_S, H_2_O_2_, and vasoactive peptides, have been suggested.

A key element in the EDH pathway supported by most studies is activation of endothelial small- and intermittent-conductance Ca^2+^-activated K^+^ channels (SK_Ca_ and IK_Ca_, resp.) upon agonist- or shear stress-induced increase of endothelial cell calcium ([Ca^2+^]_in_) [6]. Although activation of both SK_Ca_ and IK_Ca_ channels can lead to vasodilation, their contribution to the EDH is different [3–5]. SK_Ca_ channels are distributed homogeneously over the endothelial cell membrane and respond to increase of [Ca^2+^]_in_ [7, 8]. Hyperpolarization produced by the SK_Ca_ channels is believed to spread via myoendothelial gap junctions to smooth muscle cells. This original hypothesis is, however, modified [5, 9] based on the observation that SK_Ca_-dependent hyperpolarization can be blocked not only by apamin, a SK_Ca_ channel inhibitor, but also by Ba^2+^ in concentrations specifically blocking the inward-rectifying K^+^ channels (K_ir_) [10]. It has been suggested that K^+^ efflux through the opened SK_Ca_ channels might increase the local [K^+^]_out_ and consequently open K_ir_ channels. In this model the localized [K^+^]_out_ increase amplifies endothelial hyperpolarization generated by SK_Ca_ channel opening [5].

In contrast, the IK_Ca_ channels are localized in endothelial cell projections near the adjacent smooth muscle cells [11] where K^+^ efflux through these channels increases [K^+^]_out_ by about 6 mM [12], which has been called a “K^+^ cloud” [13]. The “K^+^ cloud” may hyperpolarize smooth muscle cells via activation of the Na^+^,K^+^-ATPase and K_ir_ channels [11, 14]. This hypothesis is supported by ouabain- and Ba^2+^-sensitive hyperpolarization of smooth muscles in endothelium-denuded arteries with few mM elevation of [K^+^]_out_ [12]. Both these K^+^ sensors, that is, the Na,K-ATPase and the K_ir_ channels, are expressed in smooth muscle cells [11, 15] although their importance varies between vascular beds [5].

How endothelial excitation leads to differentiated activation of SK_Ca_/IK_Ca_ channels remains unclear but this may be modulated by [Ca^2+^]_out_ via the Ca^2+^-sensing receptor (CaSR) [16]. Thus, changes in [Ca^2+^]_out_ may switch the EDH signaling between being predominantly SK_Ca_-/myoendothelial gap junction-dependent and being IK_Ca_-/“K^+^ cloud” dependent pathways.

Based on these considerations we hypothesized that modulation of the EDH-like response by [Ca^2+^]_out_ is dependent on [K^+^]_out_ and tested the importance of IK_Ca_, Na^+^,K^+^-ATPase, and K_ir_ channels for these effects of [Ca^2+^]_out_ and [K^+^]_out_.

## 2. Methods

All experiments were approved by and conducted with permission from the Animal Experiments Inspectorate of the Danish Ministry of Food, Agriculture and Fisheries. Rats were euthanized by CO_2_-inhalation.

*In vitro* functional experiments were performed on rat mesenteric small artery. Rat mesentery was dissected and placed in ice-cold physiological salt solution (PSS). Third-order branches of the rat mesenteric small artery were dissected. The cleaned arterial segments were mounted in an isometric wire myograph (Danish Myo Technology A/S, Denmark) as described previously [17]. The myograph chamber was heated to 37°C, while the PSS was constantly aerated with 5% CO_2_ in air. Force was recorded with a PowerLab 4/25-Chart7 acquisition system (ADInstruments Ltd., New Zealand) and converted to wall tension by dividing force with double segment length. Contractile concentration-response relationships were constructed by cumulative noradrenaline concentrations (NA: 0.1–30 *μ*M). The relaxation concentration-response relationships were constructed by cumulative addition of acetylcholine (ACh) (0.01–10 *μ*M) to the arteries preconstricted with 6 *μ*M noradrenaline. A maximum of three concentration-response relaxations were made on one artery.

The 4.2 mM [K^+^]-containing PSS composition was (in mM) 119.00 NaCl, 3.0 KCl, 1.18 KH_2_PO_4_, 1.17 MgCl_2_, 25.0 NaHCO_3_, 0.026 EDTA, and 5.5 glucose, gassed with 5% CO_2_ in air and adjusted to pH 7.4. The 5.9 mM [K^+^]-containing PSS composition was (in mM) 119.00 NaCl, 4.7 KCl, 1.18 KH_2_PO_4_, 1.17 MgSO_4_, 25.0 NaHCO_3_, 0.026 EDTA, and 5.5 glucose, gassed with 5% CO_2_ in air and adjusted to pH 7.4. Either 1 mM or 2.5 mM CaCl_2_ was added to the PSS as indicated. All chemicals were obtained from Sigma-Aldrich (Brondby, Denmark). Drugs were applied 15 minutes before experiment.

Statistical analyses were performed using GraphPad Prism 5 (Graph Pad Software Inc., USA). Data are expressed as mean values ± SEM. Concentration-response curves were fitted to experimental data using four-parameter, nonlinear regression curve fitting. From these curves, pD_2_ (the concentration required to produce a half-maximal response) and maximal response were derived and compared using an extra sum-of-squares *F* test. *t*-test was used where appropriate. *P* < 0.05 was considered significant. *n* refers to number of rats.

## 3. Results

**Figure 1:**
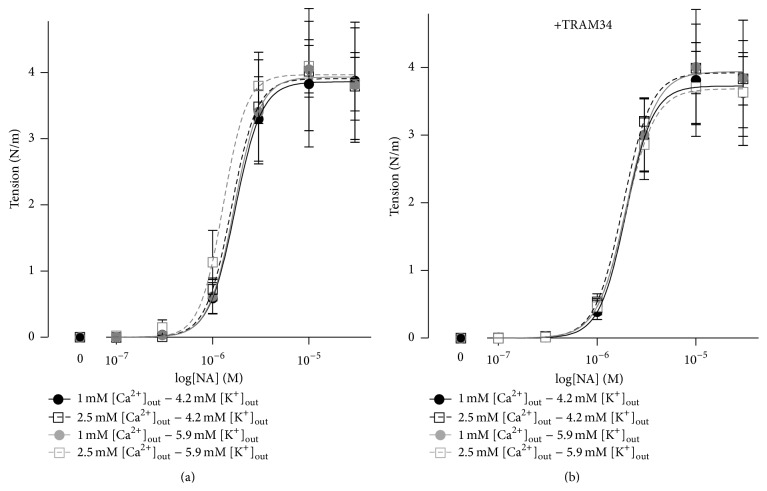
Concentration-dependent contractions to noradrenaline (NA) of rat mesenteric small arteries were not different in the presence of 1 mM and 2.5 mM [Ca^2+^]_out_. The contractions were also the same in solutions with 4.2 mM [K^+^]_out_ and with 5.9 mM [K^+^]_out_. Preincubation with 10 *μ*M TRAM34 was without significant effect on the contractile responses ((a) versus (b)). *n* = 6.

The sensitivities (pD_2_) and maximal responses to noradrenaline were the same with all four experimental solutions, that is, 1 mM and 2.5 mM [Ca^2+^]_out_ and 4.2 mM or 5.9 mM [K^+^]_out_ (Figure 1(a)). Thus, the preconstriction levels in relaxation experiments were the same in all experimental conditions. Inhibition of the IK_Ca_ channels with 10 *μ*M TRAM34 did not affect the concentration-response relationship to noradrenaline (Figure 1(b)). Neither pD_2_ nor maximal contractile responses were significantly affected by TRAM34.

**Figure 2:**
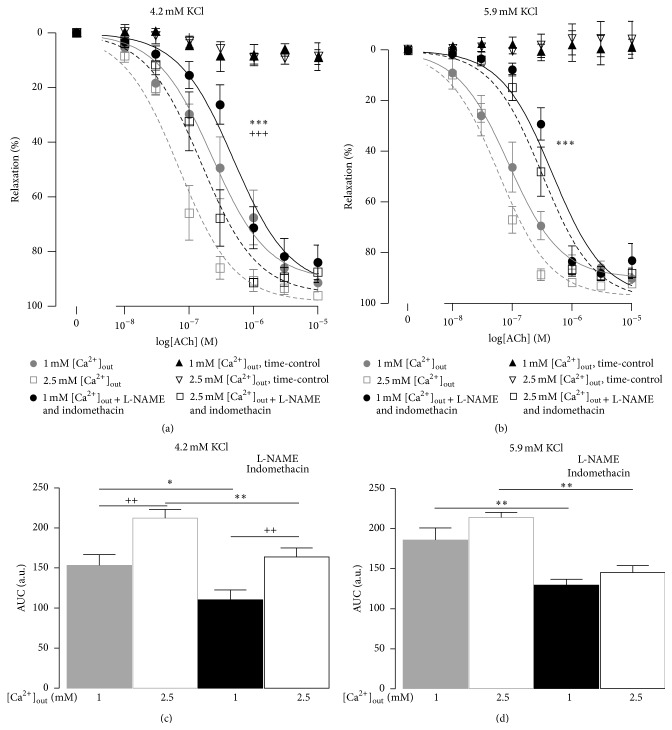
The concentration-dependent relaxation to ACh in the bath solutions containing either 4.2 mM ((a) *n* = 8) or 5.9 mM ((b) *n* = 6) [K^+^]_out_. In the time-control experiments only the vehicle was supplied (*n* = 2–4). Responses were compared under control conditions and after preincubation with 100 *μ*M L-NAME and 3 *μ*M indomethacin. (c) and (d) show the areas under curve (AUC) for the concentration-responses shown in (a) and (b). ++ and +++ indicate *P* < 0.01 and 0.001 for responses in the presence of 1 mM [Ca^2+^]_out_ versus 2.5 mM [Ca^2+^]_out_. *∗∗* and *∗∗∗* indicate *P* < 0.01 and 0.001 for responses before and after application of L-NAME and indomethacin.

Arteries preconstricted with 6 *μ*M noradrenaline were relaxed with cumulative addition of ACh (Figure 2). The preconstriction remained stable in time-control experiments where only vehicle was applied (Figures 2(a) and 2(b)).

In the 4.2 mM [K^+^]_out_ containing solution the relaxation was more pronounced when [Ca^2+^]_out_ was 2.5 mM in comparison with 1 mM [Ca^2+^]_out_ (Figures 2(a) and 2(c)). This was associated with a higher sensitivity to ACh, pD_2_  7.16 ± 0.07 versus 6.45 ± 0.09 (*P* < 0.01, *n* = 8). When [K^+^]_out_ in the bath solution was elevated to 5.9 mM, no difference between concentration-response curves at 1 mM and 2.5 mM [Ca^2+^]_out_ was observed (Figures 2(b) and 2(d)); pD_2_ were 7.06 ± 0.08 and 7.23 ± 0.05 (*n* = 6), respectively.

Preincubation of arteries with 100 *μ*M L-NAME and 3 *μ*M indomethacin significantly suppressed the relaxation to ACh in the presence of both 4.2 mM and 5.9 mM [K^+^]_out_. Importantly, these inhibitors shifted the concentration-response curves to the right (Figure 2). However, when the bath solution contained 4.2 mM [K^+^]_out_ the relaxation was still more pronounced in the presence of 2.5 mM compared to 1 mM [Ca^2+^]_out_ (Figures 2(a) and 2(c)). When [K^+^]_out_ was 5.9 mM, no difference in the relaxations at different [Ca^2+^]_out_ was seen (Figures 2(b) and 2(d)).

**Figure 3:**
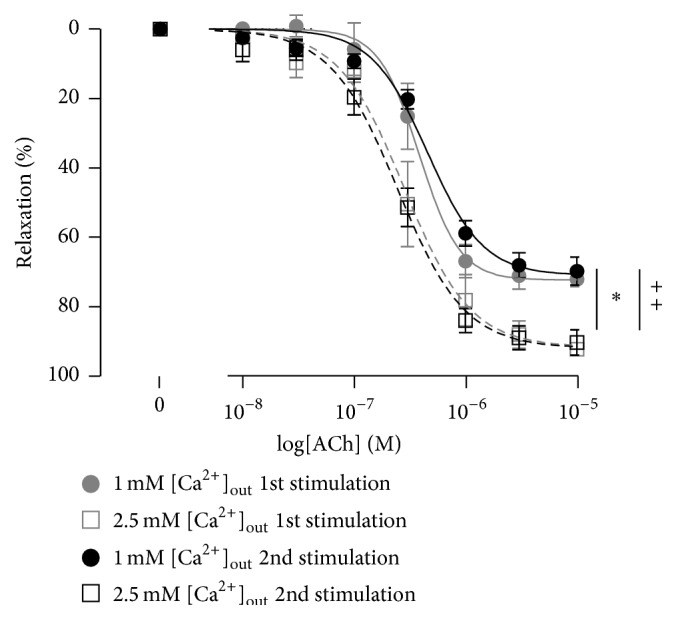
Repeated ACh concentration-relaxation curves in the presence of 100 *μ*M L-NAME and 3 *μ*M indomethacin (*n* = 5). Relaxations were first performed in the presence of either 1 mM or 2.5 mM [Ca^2+^]_out_; the bath solution was then replaced with either 2.5 mM or 1 mM [Ca^2+^]_out_. This protocol was repeated once more. *∗* indicates *P* < 0.05 for 1 mM versus 2.5 mM [Ca^2+^]_out_ for the 1st stimulations; ++ indicates *P* < 0.05 for 1 mM versus 2.5 mM [Ca^2+^]_out_ for the 2nd stimulations.

Repeated relaxations to cumulative addition of ACh in the presence of L-NAME and indomethacin were similar (Figure 3), demonstrating that there was no time-dependent effect on relaxation to ACh.

**Figure 4:**
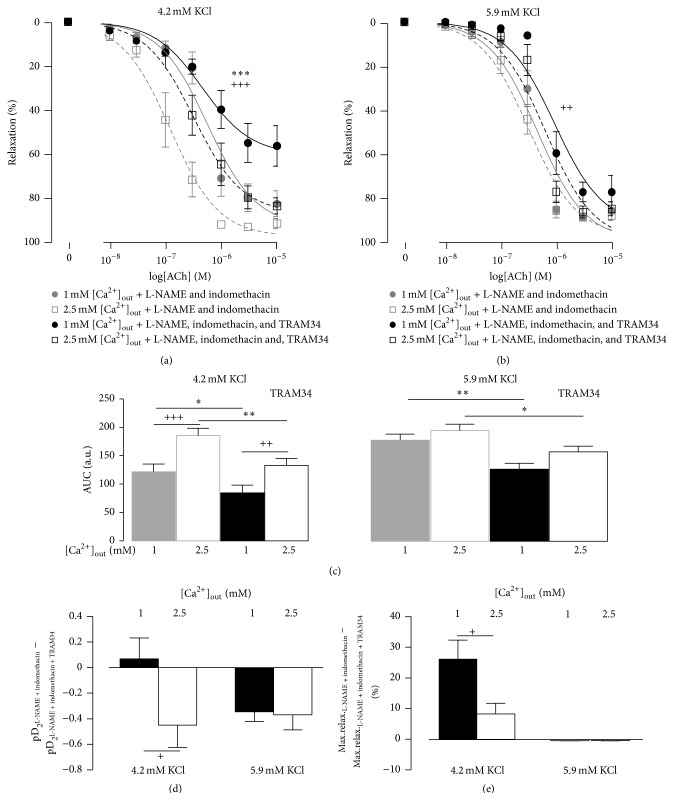
The concentration-dependent relaxation to ACh in the bath solutions containing 4.2 mM ((a) *n* = 8) and 5.9 mM ((b) *n* = 8) [K^+^]_out_. Responses were compared after preincubation with 100 *μ*M L-NAME and 3 *μ*M indomethacin (grey curves) and after preincubation with 100 *μ*M L-NAME, 3 *μ*M indomethacin, and 1 *μ*M TRAM34 (black curves). (c) shows the areas under curve (AUC) for the concentration-response curves shown in (a) and (b). (d) shows a shift in sensitivities to ACh (pD_2_) after addition of TRAM34 in the experiments shown in (a) and (b). (e) shows changes in the maximal relaxations (to 10 *μ*M ACh) for the experiments shown in (a) and (b). +, ++, and +++ indicate *P* < 0.05, 0.01, and 0.001 for responses in the presence of 1 mM versus 2.5 mM [Ca^2+^]_out_. *∗*, *∗∗*, and *∗∗∗* indicate *P* < 0.05, 0.01, and 0.001 for responses before and after incubation with TRAM34.

To assess the importance of IK_Ca_ for the effect of [Ca^2+^]_out_ we repeated the experiments in the presence of 1 *μ*M TRAM34. TRAM34 suppressed the relaxations to ACh in the presence of L-NAME and indomethacin (Figures 4(a) and 4(b)). In the 4.2 mM [K^+^]_out_ containing solution the relaxations in the presence of TRAM34 were still stronger at 2.5 mM than at 1 mM [Ca^2+^]_out_. When [K^+^]_out_ was 5.9 mM no difference in relaxations at 1 mM and 2.5 mM [Ca^2+^]_out_ was seen in the presence of TRAM34 (Figure 4(c)).

In spite of similar changes in the areas under the curve, TRAM34 affected the concentration-response curves differently at 1 mM and 2.5 mM [Ca^2+^]_out_ when [K^+^]_out_ was 4.2 mM. TRAM34 reduced the sensitivity to ACh at 2.5 mM [Ca^2+^]_out_ (Figure 4(d)) but suppressed the maximal relaxation at 1 mM [Ca^2+^]_out_ (Figure 4(e)).

At 5.9 mM [K^+^]_out_ TRAM34 had the same effect on pD_2_ to ACh in the presence of 1 mM and 2.5 mM [Ca^2+^]_out_ (Figure 4(d)). No effect of TRAM34 on maximal relaxation was seen in the presence of 5.9 mM [K^+^]_out_ (Figure 4(e)).

Preincubation of arteries with L-NAME, indomethacin, TRAM34, and apamin completely inhibited the ACh-dependent relaxations independently of [K^+^]_out_ and [Ca^2+^]_out_ (not shown, *n* = 6–8).

**Figure 5:**
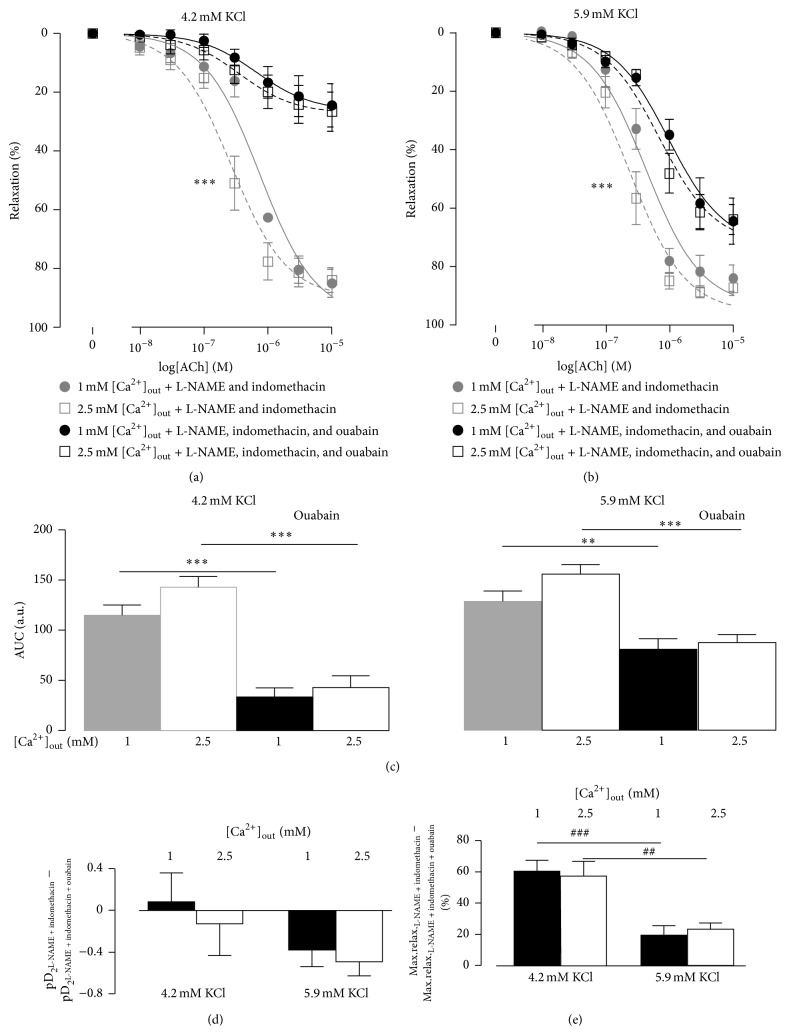
Incubation with 10 *μ*M ouabain in the presence of 100 *μ*M L-NAME and 3 *μ*M indomethacin suppressed ACh-dependent relaxations. The concentration-response curves were constructed for the experiments in the bath solutions containing 4.2 mM ((a) *n* = 8) and 5.9 mM ((b) *n* = 8) [K^+^]_out_. (c) shows the areas under curves (AUC) from (a) and (b). The changes in sensitivities to ACh (pD_2_) before and after incubation are shown in (d). The changes in maximal relaxations (to 10 *μ*M ACh) before and after the treatment with ouabain are shown in (e). *∗∗* and *∗∗∗* indicate *P* < 0.01 and 0.001 for the responses before and after incubation with ouabain. ## and ### indicate *P* < 0.01 and 0.001 for 4.2 mM versus 5.9 mM [K^+^]_out_.

To assess the importance of the Na^+^,K^+^-ATPase for the effect of [Ca^2+^]_out_ we repeated the experiments in the presence of 10 *μ*M ouabain. Ouabain suppressed the ACh-induced relaxation in the presence of 100 *μ*M L-NAME and 3 *μ*M indomethacin (Figure 5). Ouabain suppressed the relaxation more in the presence of 4.2 mM [K^+^]_out_ than in the presence of 5.9 mM [K^+^]_out_ (Figure 5(c)). In the presence of ouabain there was no difference between the areas under curves in the presence of 1 mM and 2.5 mM [Ca^2+^]_out_ at any [K^+^]_out_. Importantly, the effects of ouabain on the sensitivity to ACh and the maximal relaxations were independent of [Ca^2+^]_out_ (Figures 5(d) and 5(e)). However, ouabain suppressed the maximal relaxations at 4.2 mM [K^+^]_out_ more than in the presence of 5.9 mM [K^+^]_out_ (Figure 5(e)).

**Figure 6:**
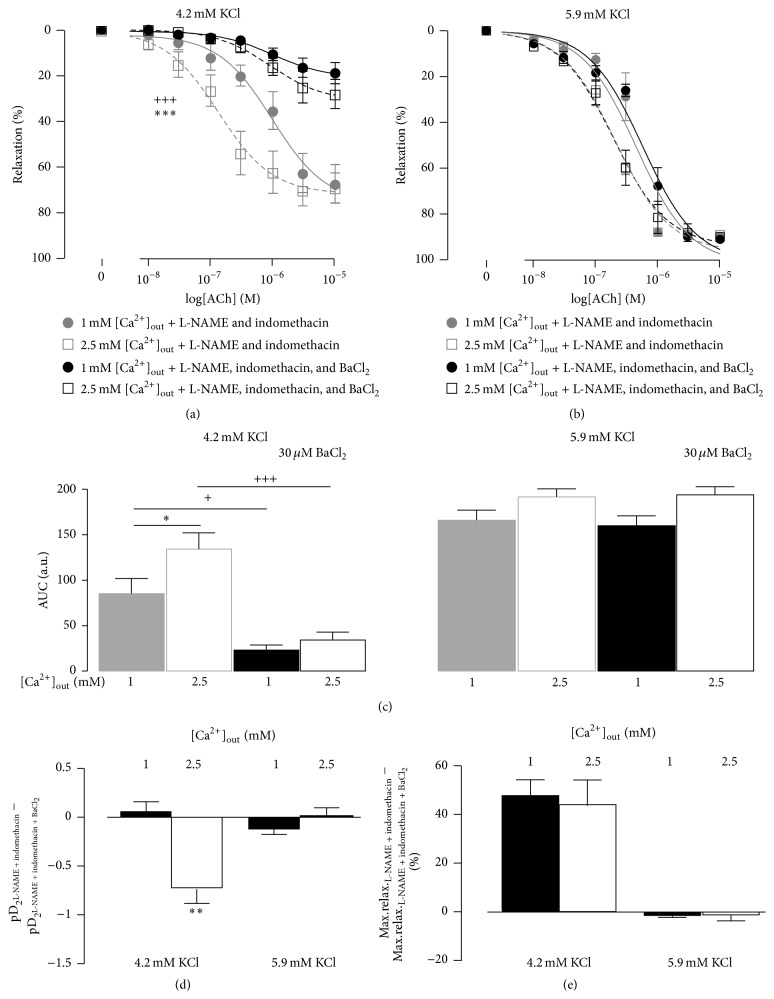
Addition of 30 *μ*M BaCl_2_ to the solution containing 100 *μ*M L-NAME and 3 *μ*M indomethacin suppressed ACh-dependent relaxations. The concentration-response curves were constructed in the bath solutions with 4.2 mM ((a) *n* = 6) and with 5.9 mM ((b) *n* = 6) [K^+^]_out_. (c) shows the areas under curves (AUC) as in (a) and (b). Changes in the sensitivities to ACh (pD_2_) after addition of BaCl_2_ are shown in (d). The effects of BaCl_2_ on the maximal relaxations are shown in (e). *∗∗* indicates *P* < 0.01 versus 1 mM [Ca^2+^]_out_.

To assess the importance of K_ir_ for the effect of [Ca^2+^]_out_ we repeated the experiments in the presence of 30 *μ*M BaCl_2_. BaCl_2_ inhibited the ACh-dependent relaxation only when the bath solution contained 4.2 mM [K^+^]_out_ (Figure 6(a)). In the presence of BaCl_2_ no differences between the areas under the curve at 1 mM and 2.5 mM [Ca^2+^]_out_ were seen (Figure 6(c)). No effect of BaCl_2_ on the relaxations at 5.9 mM [K^+^]_out_ was seen.

The effect of BaCl_2_ at 4.2 mM [K^+^]_out_ was dependent on [Ca^2+^]_out_. BaCl_2_ suppressed the sensitivity to ACh only when [Ca^2+^]_out_ was 2.5 mM (Figure 6(d)). In contrast, the maximal relaxations were similarly suppressed in the presence of both 1 mM and 2.5 mM [Ca^2+^]_out_ (Figure 6(e)).

## 4. Discussion

We have studied the [Ca^2+^]_out_-dependent modulation of EDH-like signaling and the role of IK_Ca_ and the [K^+^]_out_ sensors, that is, the Na^+^,K^+^-ATPase and K_ir_ channels, for this modulation.

### 4.1. [Ca^2+^]_out_ and the IK_Ca_ Channels

We found that an elevation of [Ca^2+^]_out_ from 1 to 2.5 mM increases the relaxation to ACh. This was also seen after the blockade of NO- and prostaglandin-dependent pathways. This is in accordance with the previous observations where wall tension and membrane potential were measured [11]. We did not measure membrane potential, but the sensitivity of NO- and prostaglandin-independent relaxation to TRAM34 and apamin suggests that this effect was mediated via an EDH-like pathway. The effects of two relatively low (1 mM) and high (2.5 mM) [Ca^2+^]_out_ concentrations were compared, because IK_Ca_ channel-dependent relaxation has previously been shown to differ strikingly at these two concentrations of Ca^2+^ [11].

Although the high [Ca^2+^]_in_ in activated endothelial cells activates both SK_Ca_ and IK_Ca_ channels, the relative importance of SK_Ca_ and IK_Ca_ has been suggested to depend on [Ca^2+^]_out_ in the myoendothelial space [7, 8]. It has been suggested that [Ca^2+^]_out_ is sensed by the G-protein-coupled CaSR [18] which can modulate the IK_Ca_ channel-dependent EDH in the vascular wall [11, 16, 19]. It was therefore tempting to speculate that the effect of [Ca^2+^]_out_ changes seen in the present study could be explained by changes in the IK_Ca_ activity. However, after inhibition of IK_Ca_ with TRAM34 there was still a modulating effect of [Ca^2+^]_out_. This suggests that the modulatory role of [Ca^2+^]_out_ cannot be limited to an effect on the IK_Ca_ channels.

Part of the Ca^2+^ effect may, however, be via the IK_Ca_ channels since TRAM34 affected the ACh concentration-response curves differently at the two [Ca^2+^]_out_ concentrations. This contrasts with a previous report [11] where TRAM34 eliminated the modulating effect of [Ca^2+^]_out_ on ACh relaxations. However, the actual values obtained in the previous study [11] were rather similar to those found in the present study; in the presence of TRAM34 the maximal relaxation tended to be less with 1 mM compared to 2.5 mM [Ca^2+^]_out_ [11]. Thus, the effect of [Ca^2+^]_out_-CaSR signaling on the IK_Ca_ channels likely explains a part of the effect of [Ca^2+^]_out_ on EDH, possibly via modulation of [Ca^2+^]_in_ [20, 21].

### 4.2. [Ca^2+^]_out_ and the Na^+^,K^+^-ATPse/K_ir_ Channels

Simultaneous inhibition of SK_Ca_ channels with apamin and IK_Ca_ channels with TRAM34 resulted in complete inhibition of endothelium-dependent relaxation. In contrast to TRAM34, the inhibitory effect of apamin was larger at 2.5 mM [Ca^2+^]_out_. It is therefore possible that [Ca^2+^]_out_ may also modify the activity of the SK_Ca_ channels. Another possibility is that [Ca^2+^]_out_ has an effect downstream for activation of the SK_Ca_/IK_Ca_ channels.

A downstream effect of [Ca^2+^]_out_ might include an effect on the two K^+^ sensors, the ouabain-sensitive Na^+^,K^+^-ATPase and K_ir_ channels, which are also known to be involved in EDH signaling [5]. To address this possibility we assessed the effect of inhibition of the Na^+^,K^+^-ATPase with ouabain [11, 22] and the effect of inhibition of the K_ir_ channels with Ba^2+^ [23, 24]. Both these interventions reduced the relaxation to ACh as expected. Importantly, in the presence of both ouabain and Ba^2+^  [Ca^2+^]_out_ had no effect on the ACh response. This might suggest that the effect of [Ca^2+^]_out_ is mediated via the Na,K-ATPase and K_ir_ channels.

Interestingly, inhibition of either the Na^+^,K^+^-ATPase or K_ir_ channels caused more than 50% inhibition of the relaxation at 4.2 mM [K^+^]_out_. If these K^+^ sensors act strictly in parallel, this is surprising. A possibility is that these transporters interact functionally and support the activity of each other. Thus, it has previously been shown that the Na^+^,K^+^-ATPase associates functionally with some K^+^ channels, for example, the ATP-dependent K^+^ channels (K_ATP_) [25]. K^+^ ions leaving the cell through the K^+^ channels have been shown to supply the Na^+^,K^+^-ATPase with K^+^ even in [K^+^]_out_-free media [26], while the Na^+^,K^+^-ATPase activity provides a gradient for the ionic current through the K^+^ channels [25]. A similar functional interaction has previously been reported in the heart [27] and pancreas [28]. It is possible that the Na^+^,K^+^-ATPase and the K_ir_ channels may also interact functionally to short-circuit the membrane K^+^ transport.

Both the Na^+^,K^+^-ATPase and K_ir_ channels are expressed in the smooth muscle cells, although they may also be present in the endothelial cells [11, 19, 29]. The effect of [Ca^2+^]_out_ might therefore be mediated via either smooth muscle or endothelial cells. Some studies reported that the effect of Ba^2+^ is endothelium-dependent [14, 30–33]. These endothelial K_ir_ channels can still be modulated by a “K^+^ cloud” thereby amplifying endothelial hyperpolarization which then spreads through the myoendothelial gap junctions. The present study was performed on endothelium-intact arteries making it impossible to distinguish the functional localization of the Na^+^,K^+^-ATPase and K_ir_ channels. But regardless of their localization our results indicate that the Na^+^,K^+^-ATPase or K_ir_ channels or both are modulated by [Ca^2+^]_out_ and that they act as sensors for [K^+^]_out_. The localized [K^+^]_out_ can act either as EDHF (in case of smooth muscle cell localization of the K^+^ sensors) or by amplifying the endothelial hyperpolarization which then spreads through the myoendothelial gap junctions (in case of endothelial localization of the K^+^ sensors).

### 4.3. The Localized [K^+^]_out_ Signaling

The importance of [Ca^2+^]_out_ was studied at two concentrations of [K^+^]_out_. We chose values close to physiological values for rats based on the observation that increase in [K^+^]_out_ to 5.9 mM induces BaCl_2_-sensitive relaxation while relaxation to [K^+^]_out_ above 5.9 mM is diminishing [13, 19, 30, 34]. In addition, it has been suggested that the Na^+^,K^+^-ATPase in the vasculature is fully saturated at 5.9 mM [K^+^]_out_ [24, 35]. This suggests that any relaxation in the presence of 5.9 mM [K^+^]_out_ is unlikely to be caused by an increase of [K^+^]_out_.

This suggestion was supported by our observation that in the presence of 5.9 mM [K^+^]_out_ ouabain and particularly Ba^2+^ had a little effect on the relaxation, while at 4.2 mM [K^+^]_out_ pronounced effects of these inhibitors were seen. This could be because the elevation of [K^+^]_out_ to 5.9 mM saturates the Na^+^,K^+^-ATPase and K_ir_ channels in agonist preconstricted arteries [19]. In fact, the “K^+^ cloud” can be generated not only by the endothelium but also via K^+^ efflux from big-conductance Ca^2+^-activated K^+^ channels which are activated in depolarized smooth muscle cells of agonist preconstricted arteries [14]. It has previously been shown that, under experimental conditions where the K^+^-sensors are saturated, inhibition of the Na,K-pump and K_ir_ channels has no effect on relaxation [14]. This indicates that the EDH under these conditions spreads through other mechanisms, possibly as a hyperpolarizing current via myoendothelial gap junctions.

Consistent with this, the modulating effect of [Ca^2+^]_out_ seen at 4.2 mM [K^+^]_out_ was abolished at 5.9 mM [K^+^]_out_. This finding is further consistent with our suggestion that the modulatory function of [Ca^2+^]_out_ is mediated largely via the Na,K-pump and K_ir_ channels. Finally, the lack of effect of TRAM34 at 5.9 mM [K^+^]_out_ is consistent with the hypothesis [11] that the importance of the IK_Ca_ channels for the EDH is associated with a localized increase in [K^+^]_out_ [12] which acts through the Na,K-pump and K_ir_ channels.

While no effect of Ba^2+^ was seen at 5.9 mM [K^+^]_out_, a small but significant inhibition of the relaxation was observed in the presence of ouabain. If at this [K^+^]_out_ the Na^+^,K^+^-ATPase is completely saturated as suggested [24, 35], it is possible that the observed effect of ouabain was mediated via modulation of gap junctions. It has previously been shown that inhibition of the Na^+^,K^+^-ATPase suppresses intercellular communications, including myoendothelial gap junctions [22, 26, 36, 37]. Thus, part of the ouabain effect can be related to inhibition of EDH spreading to smooth muscles through the myoendothelial gap junctions.

## 5. Conclusion

The main findings of this study are as follows: (i) an elevation of [Ca^2+^]_out_ enhances the EDH-like relaxation to ACh, but (ii) this Ca^2+^ effect disappears with an elevation of [K^+^]_out_. (iii) The effect of [Ca^2+^]_out_ is maintained after blocking the IK_Ca_ channels, but (iv) it disappears after blockade of the Na^+^,K^+^-ATPase and K_ir_ channels. (v) Finally, inhibitors of the Na^+^,K^+^-ATPase and K_ir_ channels, ouabain and Ba^2+^, have large effect on EDH-like relaxation only when [K^+^]_out_ is low. Thus, we have suggested that the localized [K^+^]_out_ signaling acts through the Na^+^,K^+^-ATPase and K_ir_ channels, and we have provided strong evidence that these two K^+^ sensors are affected by [Ca^2+^]_out_.
